# Effect of reduced ultrafiltration rate on the cardiac biomarkers NT-proBNP and troponin T: a prospective crossover study

**DOI:** 10.1186/s12882-026-05364-4

**Published:** 2026-09-16

**Authors:** Emelie Laveborn, Fredrik Uhlin, Nikolaos Tzimas, Anna Wärme, Ainhoa Indurain, Jan Flesche, Ellinor Bergdahl, Hans Furuland, Anders Fernström, Maria K. Svensson, Bernd Stegmayr, Michael Ott

**Affiliations:** 1 Department of Public Health and Clinical Medicine, Umeå University, Umeå, 901 87 Sweden; 2 Department of Nephrology and Department of Health, Medicine and Caring Sciences, Linköping University, Linköping, Sweden; 3 Department of Health Technologies, Tallinn University of Technology, Tallinn, Estonia; 4 Department of Medical Sciences, Renal Medicine, Uppsala University Hospital, Uppsala, Sweden; 5 Department of Nephrology, Skaraborg Hospital, Skövde, Sweden; 6 Department of Internal Medicine and Clinical Nutrition, Institute of Medicine, Sahlgrenska Academy, University of Gothenburg, Gothenburg, Sweden; 7 Department of Medical Sciences, Renal Medicine, Uppsala University, Uppsala, Sweden; 8 Uppsala Clinical Research Center, Uppsala, Sweden

**Keywords:** Haemodialysis, Heart, NT-proBNP, Troponin T, Ultrafiltration rate

## Abstract

**Background:**

High interdialytic weight gain (IDWG) and ultrafiltration rate have been associated with increased mortality in patients undergoing haemodialysis. Cardiac biomarkers reflecting strain, including N-terminal pro-B-type natriuretic peptide (NT-proBNP) and high-sensitivity troponin T (TnT), are known to increase during haemodialysis sessions. Previous studies suggest that these intradialytic increases may be related to ultrafiltration rate, with a proposed threshold of 0.6 IDWG per hour. This study therefore aimed to prospectively evaluate whether reducing ultrafiltration rate attenuates the haemodialysis-associated increase in these cardiac biomarkers.

**Method:**

In this multicentre, prospective study prevalent haemodialysis patients with a relative IDWG > 2.5% of body weight and ultrafiltration rate ≥ 0.72 IDWG per hour underwent one standard and one prolonged haemodialysis session with a ≥ 20% reduction in ultrafiltration rate, targeting an ultrafiltration rate ≤ 0.6 IDWG per hour. NT-proBNP and TnT were analysed directly before dialysis, at 180 minutes, and at the end of each haemodialysis session.

**Results:**

Of 238 screened patients, 65 met the inclusion criteria and none of the exclusion criteria. Of these, 56 were enrolled and 41 included in the final analysis, meeting the pre-specified sample size required to detect a clinically relevant difference. NT-proBNP and TnT increased significantly during both dialysis sessions (*p* < 0.001). However, no difference was observed in ∆NT-proBNP (*p* = 0.967) or ∆TnT (*p* = 0.823) between treatments with different ultrafiltration rates. Achieving an ultrafiltration rate ≤ 0.6 IDWG per hour required a mean treatment duration of 423 ± 139 minutes (min 246, max 775). Among the 12 patients (29.3%) who achieved the targeted ultrafiltration rate of ≤0.6 IDWG per hour, changes in NT-proBNP (*p* = 0.062) and TnT (*p* = 0.114) did not differ significantly between sessions with different ultrafiltration rates.

**Conclusions:**

In stable, prevalent haemodialysis patients who tolerate their ordinary treatment, reducing the ultrafiltration rate by at least 20% did not attenuate the increase in NT-proBNP and TnT during a single haemodialysis session. In these patients, achieving an ultrafiltration rate ≤ 0.6 IDWG/h generally required treatment durations that are not feasible in routine in-centre haemodialysis.

**Trial registration:**

Registered at clinicaltrials.gov (NCT06153888) November 6 2023 (Retrospectively registered).

**Supplementary Information:**

The online version contains supplementary material available at https://doi.org/10.1186/s12882-026-05364-4.

## Introduction

The prevalence of cardiovascular disease (CVD) rises as kidney function declines and remains a leading cause of morbidity and mortality in patients receiving haemodialysis [1–3]. These patients have a substantial burden of traditional, but also uraemia-specific, cardiovascular risk factors, such as anaemia, fluid overload, retention of uraemic toxins, and disturbances in calcium-phosphate balance, all of which promote CVD [4].

Haemodialysis itself contributes to structural and functional changes in the heart. Repeated episodes of intradialytic hypotension, reduced myocardial blood flow, and myocardial stunning can, over time, lead to permanent myocardial injury [5]. Such injury is thought to contribute to the development of myocardial fibrosis [6] and the increase in left ventricular mass index (LVMI) observed in many individuals undergoing long-term haemodialysis [7].

Overhydration is associated with impaired left ventricular diastolic function [8], increased LVMI [9], and mortality [10]. High inter-dialytic weight gain (IDWG) is associated with an increased risk of cardiac events [11] and death [12]. To manage fluid overload, ultrafiltration is required during haemodialysis. High IDWG often necessitates higher ultrafiltration rates, which have been linked to an increased mortality in several large retrospective cohort studies [13–17]. The risk appears to rise already from ultrafiltration rates as low as 6 ml/h/kg [16]. Imaging studies using magnetic resonance imaging (MRI) or echocardiograms suggest that the acute cardiac effects of haemodialysis and the extent of the functional and structural damages seen during a haemodialysis session correlated with increased ultrafiltration rate [18, 19].

N-terminal-pro-B-type natriuretic peptide (NT-proBNP) and high-sensitivity cardiac Troponin T (TnT) are well-established markers of cardiac strain and injury [20] and have been linked to increased mortality [21–23]. NT-proBNP release is induced by stretching of the myocardial wall, often as a response to volume overload [24] whereas TnT mainly is released following myocardial cell damage [25]. Levels of both markers increase during haemodialysis, even after correction for haemoconcentration [26], particularly in patients with IDWG over 2.5% of body weight [27]. However, the increase in cardiac markers during haemodialysis is masked when using high flux dialysers, making it necessary to use low flux dialysers for study purposes [26]. In a study using high flux dialysers, a lower reduction during haemodialysis was a risk factor for mortality [28]. Even in healthy individuals, small, acute increases in TnT affects mortality risk [29]. A previous study found a positive correlation between the ultrafiltration rate and the increase in NT-proBNP during a single haemodialysis session, defining a breaking point of the ultrafiltration rate relative to body weight exceeding 0.6 IDWG/h [30].

Previous studies examining the effects of different ultrafiltration rates have been largely observational and retrospective in their nature, limiting the ability to isolate the impact of ultrafiltration rate from other patient-related factors. Using a prospective, crossover design in which patients serve as their own controls, minimizes these confounders.

The aim of this study was to determine whether reducing the ultrafiltration rate, by longer dialysis, attenuates the increase in NT-proBNP and TnT seen during a haemodialysis session.

## Materials and methods

The study was a multicentre, prospective, self-controlled crossover study (NCT06153888 registered at clinicaltrials.gov November 6 2023) conducted at five haemodialysis centres in Sweden. Chronic haemodialysis patients were enrolled between January 2023 and October 2025. Ethical approval was obtained from the Swedish Ethical Review Authority (2012–42-31 M, revised Dnr 2023–06921-02) and complied with the Declaration of Helsinki. All participants provided written informed consent. The study is reported in accordance with the CONSORT guidelines.

### Patient selection

All patients at the participating sites were screened. In this study, IDWG was defined as interdialytic weight gain relative to target weight. Inclusion criteria were IDWG of more than 2.5% and a relative ultrafiltration rate over 0.72 IDWG/h. Eligible patients were to perform one standard dialysis and one prolonged haemodialysis session with a ≥ 20% reduction in ultrafiltration rate, targeting an ultrafiltration rate ≤ 0.6 IDWG per hour.

Patients were excluded if they were under 18 years of age, had cognitive impairment preventing them from providing written, informed consent, used single-needle-dialysis, had medical reasons why they could not undergo dialysis treatment with a low flux dialyser or were clinically unstable (terminally ill, suffering from acute infection or anything else making it unlikely for them to keep their regular dialysis schedule without significant changes during the study period).

### Variables

NT-proBNP and TnT were used as markers for cardiac strain. Background variables included age, sex, renal diagnosis, dialysis vintage, comorbidities, ordinary dialysis regimen, haemodialysis access, weight, and height. Pulse and blood pressure were recorded during treatment. Clotting of the dialyser was recorded and graded as clear, some degree of residual clotting, or coagulated. Adverse events were defined as symptomatic intradialytic hypotension (patient complaints of symptoms associated with hypotension in the presence of drop of blood pressure or hypotension needing intervention), intradialytic muscle cramps, and complete coagulation resulting in interruption of treatment.

From echocardiographs, left ventricular ejection fraction (EF), left ventricular dimensions, diastolic function, and aortic- and mitral valve dysfunctions were recorded. From electrocardiograms (ECG), QRS-duration was recorded.

### Outcomes

The main outcome was the difference of the change (∆) in NT-proBNP- and TnT-levels during haemodialysis sessions with ordinary ultrafiltration rate compared with reduced ultrafiltration rate.

Secondary outcomes included correlations between the primary outcomes and LVMI, EF, valve dysfunctions, QRS duration, and comorbidities as well as adverse events during haemodialysis.

### Samples size calculation

Data from Goto et al. [30] was used for power calculation. Based on clinical experience, an effect size of 0.6 was deemed clinically relevant for the difference in change of NT-proBNP during a haemodialysis session. Using two-tailed paired t-test, this would require 40 patients to achieve a power of 95% at 0.05 significance level.

### Study design

Each patient underwent two haemodialysis sessions, the same day of the week, in two consecutive weeks. Whether the study dialyses were performed after the long or the short interval (mid-week) depended on the day the patient was most likely to reach their target weight and still fulfilled the inclusion criteria. For a more precise calculation of dialysis session duration, it was preferred that the standard session was performed during week one and the prolonged session during week two. However, if this was not possible because of scheduling difficulties, the standard session could instead be performed one week after the prolonged session. One haemodialysis session was performed with the ordinary dialysis prescription, and one session with prolonged time and reduced ultrafiltration rate. To minimise confounding, the subsequent session within the study was planned one week later with change of only the treatment time. All other aspects of the dialysis prescription (dialyser, blood flow, dialysate flow, temperature, and dialysate composition) were kept unchanged. Between the two sessions, target weight and blood pressure medications were not altered.

In the case of prolonged session, the principal investigator at each site was free to increase the dose of anticoagulation as deemed appropriate to reduce the risk of clotting.

All haemodialysis sessions were performed with low flux dialysers (FX10® or FX8®, Fresenius Medical Care GmbH, Bad Homburg, Germany).

Target weight was adjusted before the first study session to ensure accurate target weight. This was done according to clinical practice at the participating sites.

### Determination of treatment time

Standard dialysis time was based on routine prescription. The study dialysis was prolonged to achieve a ≥ 20% reduction in ultrafiltration rate compared with the standard dialysis. If possible, an ultrafiltration rate of ≤0.6 IDWG/h was targeted. This approach was based on findings from a previous study indicating that the increase in NT-proBNP differed between patients with ultrafiltration rates above versus below this threshold [30]. In all cases, the selected duration approximated the target as closely as feasible within operational constraints. The calculation formula is provided in Supplementary Item 1.

### Data collection

Blood samples for analysis of NT-proBNP, TnT, urea and haematocrit were drawn before treatment start, after 180 minutes, and five minutes before the end of treatment. The blood samples were immediately sent to the local laboratory for analysis. NT-proBNP levels above the upper limit of quantitation were diluted until quantifiable measurements could be made. Cardiac biomarkers taken during or at the end of haemodialysis were corrected for haemoconcentration using Schneditz formula for plasma components [31].

Pulse and blood pressure were taken using the automatic cuff linked to the dialysis machine at the same time points as the blood samples. The attending nurse clinically evaluated the pulse as regular or irregular. Parameters regarding the haemodialysis treatment, including adverse events, were recorded during treatment by the attending nurse.

Background data were collected by the principal investigator at each unit.

Echo- and electrocardiograms were considered for this study when they were performed under stable conditions within the last year. LVMI was calculated using the formula of Teichholz [32] for left ventricular mass (LVM) and indexed to body surface area.

## Statistical analysis

The effects of different ultrafiltration rates were assessed according to treatment group, standard vs. prolonged dialysis session. The analysis regarding cardiac markers during or after session was based on values adjusted for haemoconcentration.

Data were reported as mean and standard deviation (SD), median and interquartile range (IQR) or frequencies (%) as appropriate. Statistical analysis was performed using Student’s paired t-test for comparison between treatments or Wilcoxon signed rank test for not normally distributed data. Spearman´s rank correlation coefficient was calculated for non-normally distributed data. Linear mixed models were fitted to log-transformed NT-proBNP and TnT values, with treatment (short vs. long session), timepoint (baseline, 180 min, end), and their interaction as fixed effects, and a random intercept for patients. Fixed effects were tested using Type III tests with Satterthwaite degrees of freedom. Significance level was set to *p* < 0.05.

All statistical analyses were made using IBM SPSS Statistics for Windows version 30.0.0.0 (IBM Corp., Armonk, NY, USA).

## Results

**Fig. 1:**
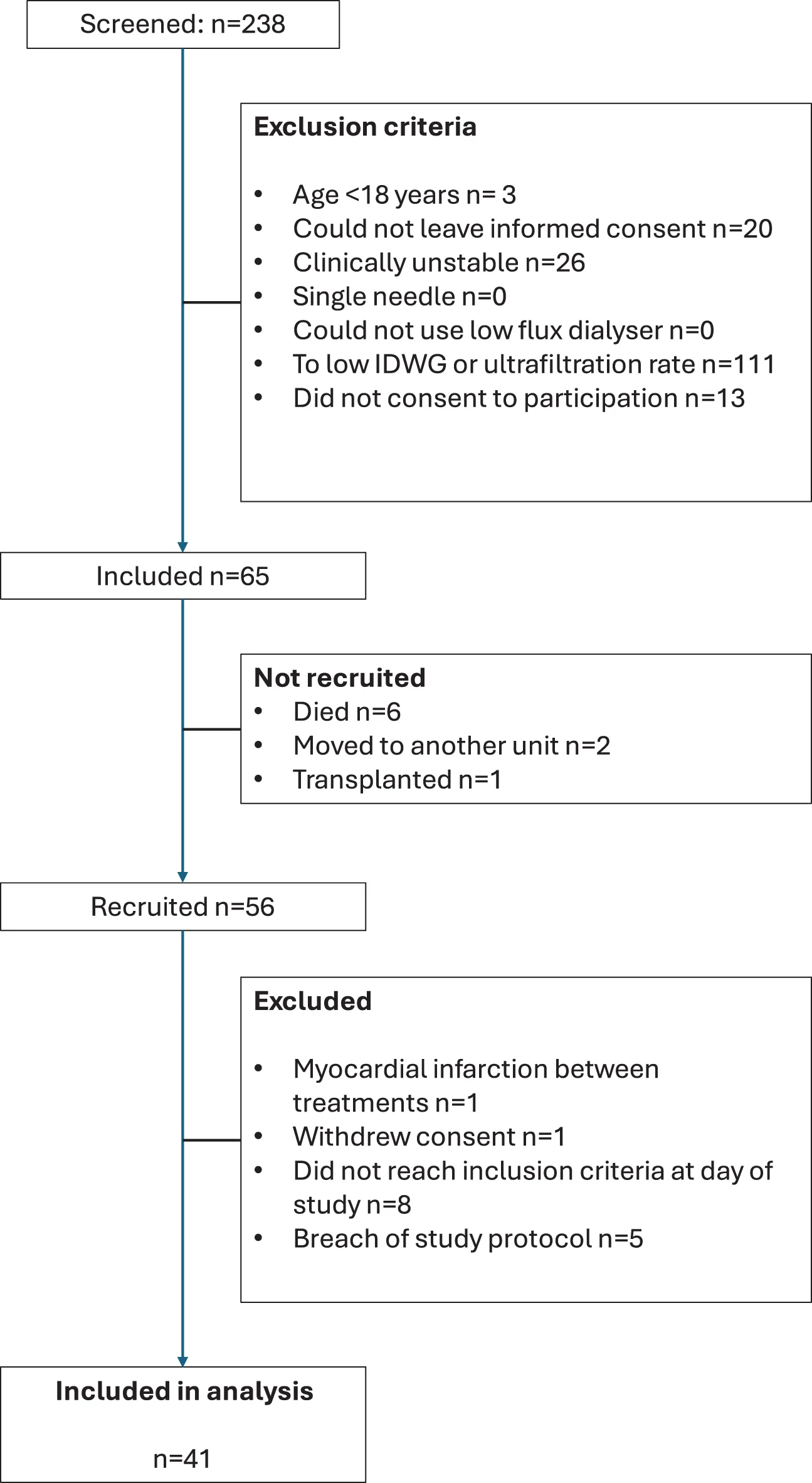
Study flow

A total of 238 patients were screened, of whom 65 patients provided informed consent and were initially enrolled. At one site, nine patients were lost prior to recruitment. During the study, an additional 15 patients were excluded, leaving 41 patients for the final analysis. The study flow-chart is presented in Fig. 1.

Baseline characteristics are presented in Table 1. All patients achieved a reduction of the ultrafiltration rate of ≥20% compared to the standard session. For one patient, NT-proBNP at the end of the treatment was missing. One site (*n* = 8) had no TnT available for analysis. There was a significant increase in NT-proBNP and TnT during both sessions (*p* < 0.001 for NT-proBNP and TnT during both sessions). Levels of cardiac markers and change during sessions are presented in Table 2. When comparing sessions with ordinary and prolonged treatment time, the difference in ∆NT-proBNP was 37.3 (IQR 1240) ng/l and the difference in ∆TnT was −0.4 (IQR 12.7) ng/l (*p* = 0.967 for NT-proBNP and *p* = 0.823 for TnT). When comparing measurements at 180 minutes and end of treatment there was a continued increase in NT-proBNP during both sessions (*p* < 0.001 for ordinary treatment and *p* = 0.002 for prolonged treatment). There was no difference in ∆TnT when comparing measurements at 180 minutes and end of treatment (*p* = 0.114 for ordinary treatment and *p* = 0.228 for prolonged treatment). Details are presented in Table 2.

**Table 1:** Baseline characteristics

Age (years)	63.5	SD 14.8
Vintage (months)	32.9	IQR 44.5
Height (cm)	170.9	SD 9.5
Weight (kg)	74.5	SD 13.8
BMI (kg/m^2^)	25.7	SD 5.4
**Sex**		
*Men*	28	68.3%
*Women*	13	31.7%
**Primary kidney diagnosis**		
*Diabetes nephropathy*	14	34.1%
*Glomerulonephritis*	10	24.4%
*Other*	7	17.1%
*Unknown*	3	7.3%
*Polycystic kidney disease*	3	7.3%
*Tubulointerstitial nephritis*	2	4.9%
*Nephrosclerosis*	2	4.9%
**Comorbidities**		
Previous myocardial infarction	7	17.1%
Persistent or permanent atrial fibrillation	6	14.6%
Pacemaker	2	4.9%
Previous stroke	10	24.4%
Diabetes mellitus	18	43.9%
*Type 1*	8	19.5%
*Type 2*	10	24.4%
Haemodialysis access**		
Central venous catheter	28	68.3%
Forearm arteriovenous fistula	10	24.4%
Upper arm arteriovenous fistula	3	7.3%
Arteriovenous graft	2	4.8%
**Ordinary treatment regimen**		
Haemodiafiltration	28	68.3%
Haemodialysis, high flux dialyser	12	29.3%
Haemodialysis, medium cut-off dialyser	1	2.4%
Treatments per week		
*One*	1	2.4%
*Two*	1	2.4%
*Three*	32	78.0%
*Four*	7	17.1%
Time per treatment (minutes)	246	SD 23.0

Data presented as mean (SD), median (IQR) or number (%)

** 2 patients had simultaneous use of catheter and functioning arteriovenous fistula

**Table 2:** Differences between treatments

	Standard session		Prolonged session		p
	Median	IQR	Median	IQR	
NT-proBNP before treatment (baseline) (ng/l)	5850.0	20950.0	4370.0	22467.0	0.789
∆NT-proBNP start-180 min of treatment (ng/l)*	784.2	4022.3	1141.1	3202.2	0.936
∆NT-proBNP start-end of treatment (ng/l)*	2244.0	6342.0	1468.9	3961.7	0.967
Tn T before treatment (baseline) (ng/l)	70.0	44.0	69.0	49.0	0.322
∆TnT start-180 min of treatment (ng/l)*	5.4	8.4	5.6	11.8	0.348
∆TnT start-end of treatment (ng/l)*	5.0	8.6	4.4	9.1	0.823
	Mean	SD	Mean	SD	p
Interdialytic weight gain (%)	4.3	1.3	4.1	1.3	0.342
Expected ultrafiltration (l)	3.1	0.9	3.2	0.8	0.842
Delivered treatment time (min)	247	25	334	37	<0.001
Ultrafiltration rate (IDWG/h)	0.97	0.18	0.75	0.21	<0.001
Kt/V	1.46	0.27	1.90	0.50	<0.001
Total dose of low molecular heparin (IE)	4506.2	1829.7	6032.5	2614.8	<0.001
Haematocrit before treatment (%)	34.2	3.7	34.0	4.0	0.652
Haematocrit after 180 min of treatment (%)	35.6	4.0	34.4	4.0	<0.001
Haematocrit at end of treatment (%)	36.2	4.0	36.0	4.1	0.352
Systolic blood pressure before treatment (mmHg)	149.0	21.9	146.6	20.0	0.181
Systolic blood pressure after 180 min of treatment (mmHg)	128.3	19.9	127.6	18.4	0.905
Systolic blood pressure at end of treatment (mmHg)	128.8	17.0	131.3	21.0	0.521
Diastolic blood pressure before treatment (mmHg)	75.7	20.0	76.0	13.5	0.971
Diastolic blood pressure after 180 min of treatment (mmHg)	70.0	12.1	69.6	13.6	0.924
Diastolic blood pressure at end of treatment (mmHg)	72.0	13.8	70.0	13.0	0.294
Pulse before treatment (bpm)	69.6	11.5	69.5	9.6	0.951
Pulse after 180 min of treatment (bpm)	68.6	10.9	68.2	8.1	0.750
Pulse at end of treatment (bpm)	68.8	10.6	71.1	15.6	0.353
	n/N	%	n/N	%	p
Symptomatic hypotension	2/33	6.1	4/33	12.1	0.500
Cramps	6/33	18.2	6/33	18.2	1
Dialyser appearance after treatment					
*Clear*	4/41	9.8	4/41	9.8	1
*Some degree of clotting*	37/41	90.2	37/41	90.2	1
*Coagulation *	0	0	0	0	1

Comparison between haemodialysis sessions with standard treatment time and prolonged treatment time. NT-proBNP: N-terminal proB-Type natriuretic peptide. TnT: High sensitivity Troponin T. *Values at 180 minutes and end of treatment are adjusted for haemoconcentration

In the linear mixed models, the treatment × timepoint interaction was not statistically significant (F (2,198) = 0.001, *p* = 0.99 for NT-proBNP, F(2,160) = 0.57, *p* = 0.56 for TnT), indicating no evidence that the trajectory of cardiac biomarkers over the session differed between short and long dialysis. There was a significant main effect of timepoint (F(2,198) = 49.1, *p* < 0.001 for NT-proBNP, F(2,160) = 15.0, *p* < 0.001 for TnT), reflecting a biomarker increase from baseline to end of session in both groups, but no significant main effect of treatment (F(1,198) = 0.44, *p* = 0.50 for NT-proBNP and F(1,160) = 2.23, *p* = 0.137 for TnT). The 95% CI derived from estimated marginal means for the interaction between ultrafiltration rate and time of measurement is presented in Supplement Table 1 (NT-proBNP) and Supplement Table 2 (TnT).

Unadjusted values are presented in Supplement Table 3. Individual values are presented in Supplement Figure 1 (NT-proBNP) and Supplement Figure 2 (TnT).

To achieve an ultrafiltration rate below 0.6 IDWG/h, a mean time of 423 ± 139 (min 246, max 775) minutes was needed. The maximum available time slot for a treatment was limited to 6–6.5 hours due to practical constrains in the haemodialysis units. A total of 12 patients (29.3%) reached an ultrafiltration rate below or equal to 0.6 IDWG/h. When analysing only these patients, there was no difference in the change of cardiac biomarkers (*p* = 0.062 for ∆NT-proBNP and *p* = 0.114 for ∆TnT). Among the 12 patients, only one demonstrated a clinically meaningful attenuation in the rise of NT-proBNP (>25 percentage points) during the prolonged session relative to the standard session.

One patient performed the prolonged session before the standard session.

In addition to clinical examination target weight was determined with the guidance of blood volume monitoring in one (2.4%) patient, NT-proBNP in 13 (31.7%) patients and echocardiography in one (2.4%) patient. Chest X-ray or lung ultrasound were not used. Bioimpedance measurement was used in 32 (78%) patients. In these patients the mean difference between target weight and normohydrated weight according to bioimpedance measurements was 1.5 ± 3.8 kg.

Echocardiographs were available for 19 patients (46.3%), of whom 14 (34.1%) had information on left ventricular measurements. Only a small number of patients exhibited more than mild valve dysfunctions. Details are presented in Supplement Table 4. Due to the small numbers in subgroups, no statistical testing was performed. However, individual data did not reveal any clear pattern suggesting subgroup differences.

An ECG was available in 40 patients of whom 10 had a QRS duration over 120 ms. Comparisons between patients with QRS < 120 ms vs ≥ 120 ms showed no difference in ∆NT-proBNP (*p* = 0.092) or ∆TnT (*p* = 0.445). Among patients with QRS ≥ 120 ms, there was no significant difference in biomarker changes between sessions with ordinary vs reduced ultrafiltration rate (*p* = 0.575 for ∆NT-proBNP, *p* = 0.779 for ∆TnT).

There was a positive correlation between age and ∆NT-proBNP during standard (*R* = 0.437, *p* = 0.005) and prolonged (*R* = 0.371, *p* = 0.018) sessions. Age also correlated with baseline levels of both cardiac markers (*R* = 0.477, *p* = 0.002 for NT-proBNP and *R* = 0.396, *p* = 0.023 for TnT). No correlation was observed between LVMI and ∆NT-proBNP (*R* = 0.253, *p* = 0.405 for ordinary treatment, *R* = 0.220, *p* = 0.471 for prolonged treatment). Detailed correlation data are presented in Supplement Table 5.

The number of adverse events was low during both treatments (Table 2).

## Discussion

In this crossover study, we investigated whether reducing the ultrafiltration rate modifies haemodialysis-induced increase in cardiac biomarkers. We found no difference in the changes of NT-proBNP and TnT between treatments with higher vs lower ultrafiltration rates. Consistent with previous observations, both NT-proBNP and TnT increased during haemodialysis sessions.

To our knowledge, this is the first prospective study to intentionally modify ultrafiltration rate in haemodialysis patients. Acute cardiac effects of haemodialysis, including myocardial stunning and the development of regional wall motion abnormalities, have been previously demonstrated [33]. Ultrafiltration has been shown to reduce cardiac output and increases systemic vascular resistance even in patients without previous cardiovascular disease [34]. Observational studies suggest a correlation between ultrafiltration rate and both functional cardiac changes [18, 19] and mortality [13–16].

Ultrafiltration rate is closely linked to IDWG, as accumulated fluid must be removed within a limited timeframe. Since most previous studies are observational, it remains unclear whether the adverse outcomes are directly attributable to high ultrafiltration rates or reflect volume overload. This is supported by previous findings indicating that persistent overhydration over time rather than dialysis frequency is associated with cardiac structural changes [9].

Patient characteristics may modify the haemodynamic impact of ultrafiltration. Previous data have suggested that age [35] and body size [36] may influence vulnerability. In the present study, only age was significantly correlated with ∆NT-proBNP. Although higher ultrafiltration rates have been linked to worse outcomes in patients with cardiac enlargement [37] we found no evidence that baseline NT-proBNP or TnT modified biomarker responses. There was no correlation between change in cardiac biomarkers and LVMI, however the number of available echocardiographs were limited.

A clear separation in ultrafiltration rates between treatments was achieved with target levels comparable to previously reported thresholds (6 ml/h/kg) [16]. Notably, half of the screened patients did not meet the inclusion criteria due to low IDWG or ultrafiltration rate during ordinary treatment, suggesting that high ultrafiltration rates are already avoided in clinical practice. Dialysis prescriptions were individualised across centres, with treatment frequency ranging from one to four sessions per week. Adverse events were infrequent, likely reflecting clinical optimisation of ultrafiltration rates to rates well tolerated by each patient. Intradialytic hypotension is a risk factor for mortality [38] and reducing ultrafiltration rates to avoid hypotension is probably beneficial, if this effects biomarker response is however unclear.

Only one third of participants reached the predefined ultrafiltration rate (≤0.6 IDWG/h) during prolonged sessions. In this subgroup, a tendency towards a smaller increase in NT-proBNP was noted, although this was clinically meaningful only for one patient. Determining ultrafiltration rate in dialysis patients needs to be individualised. A prolongation of dialysis time might be beneficial for some patients. However, for most patients, the treatment duration required to reach an ultrafiltration rate below 0.6 IDWG/h was not feasible in standard in-centre haemodialysis.

Previous singular centre data suggest improved survival with long dialysis sessions, although this may reflect centre-specific factors, including lower ultrafiltration rate and lower blood pump flow [39]. A randomized controlled trial has shown that that increased dialysis frequency may reduce LVMI [40], although this effect was not shown with nocturnal dialysis [41]. Increased dialysis intensity has also been associated with reduction in regional wall motion abnormalities [18]. These treatment modalities were not evaluated in our study.

The adverse effects of haemodialysis are likely multifactorial. Prolonged treatment duration leads to a prolonged exposure to the extracorporeal circuit, which may offset potential benefits of reducing ultrafiltration rate through a prolonged blood-membrane interaction inducing complement activation and coagulation [42], and exposure to microbubbles [43]. In our study no differences in cardiac marker trajectory were observed at 180 minutes between treatment arms. NT-proBNP continued to increase beyond 180 minutes during both sessions.

In clinical practice, reducing the ultrafiltration rate is most effectively achieved by limiting IDWG. However, this remains difficult and requires a comprehensive, patient-centred approach [44].

### Limitations

This study has several limitations. The dialysis population is highly heterogeneous; however, a crossover design was used to minimise confounding. While we reached our prespecified sample size, the ultrafiltration rate target was only achieved in few patients. While the absence of differences in marker response suggests the main findings are robust, the study was underpowered for subgroup analyses.

Since lower ultrafiltration rate with unchanged IDWG can only be achieved by prolonging treatment time, this intervention cannot fully isolate the physiological effects of ultrafiltration rate itself. However, there were no differences in change of cardiac biomarkers at 180 minutes. Since both cardiac biomarkers are dialysable with high flux dialysers and even more so with haemodiafiltration it was necessary to use low flux dialysers for this study. To evaluate treatments using haemodiafiltration, other markers or imaging techniques would be required.

Cardiac effects were assessed using biomarkers alone to assess cardiac strain. Although NT-proBNP and TnT are easily available and prognostically relevant [45], they may not capture all aspects of dialysis-induced cardiac stress. A multimodal approach incorporating imaging (echocardiographs or magnetic resonance imaging) and continuous ECG monitoring would have strengthened the analysis.

The observed increase in TnT during haemodialysis was well below the threshold for myocardial infarction. However, TnT may increase in response to myocardial stress or transient ischemia [25, 46] and peak levels may occur after the dialysis session. Therefore, peak TnT levels may not have been captured. Nevertheless, intradialytic increases in TnT have been associated with impaired myocardial function [47]. In contrast, NT-proBNP is released more rapidly, and is expected to peak during or shortly after dialysis [48].

Echocardiographic data was only available for half of the study population and were collected retrospectively. Prospective and standardized echocardiographic assessment would have provided more robust mechanistic insights and is suggested for future studies.

## Conclusion

In stable, prevalent haemodialysis patients who tolerate their ordinary treatment, reducing the ultrafiltration rate by at least 20% did not attenuate the increase in NT-proBNP and TnT during a single haemodialysis session. In these patients, achieving an ultrafiltration rate ≤ 0.6 IDWG/h generally required treatment durations that are not feasible in routine in-centre haemodialysis.

## Electronic supplementary material

Below is the link to the electronic supplementary material.

Supplementary material 1

## Data Availability

Due to its sensitive nature, data is not publicly available. Data may be available from the corresponding author upon reasonable request and after ethical review.

## Abbreviations

CVD: Cardiovascular disease
LVM: Left ventricular mass
LVMI: Left ventricular mass index
EF: Ejection fraction
NT-proBNP: N-terminal pro-B-type natriuretic peptide
TnT: hs cardiac Troponin T
IDWG: Interdialytic weight gain

## References

[1] Stel VS, Boenink R, Astley ME, Boerstra BA, Radunovic D, Skrunes R, et al. A comparison of the epidemiology of kidney replacement therapy between Europe and the United States: 2021 data of the ERA registry and the USRDS. Nephrol Dial Transpl. 2024;39(10):1593–603. 10.1093/ndt/gfae040. PMC11483573 38439701

[2] Boerstra BA, Boenink R, Astley ME, Bonthuis M, Abd ElHafeez S, Arribas Monzon F, et al. The ERA registry annual report 2021: a summary. Clin Kidney J. 2024;17(2):sfad281. 10.1093/ckj/sfad281. PMC11024806 38638342

[3] Minciunescu A, Genovese L, DeFilippi C. Cardiovascular alterations and structural changes in the setting of chronic kidney disease: a review of cardiorenal syndrome type 4. SN Compr Clin Med. 2022;5(1):15. 10.1007/s42399-022-01347-2. 36530959 PMC9734879

[4] Ronco C, Haapio M, House AA, Anavekar N, Bellomo R. Cardiorenal syndrome. J Am Coll Cardiol. 2008;52(19):1527–39. 10.1016/j.jacc.2008.07.051. 19007588

[5] Burton JO, Jefferies HJ, Selby NM, McIntyre CW. Hemodialysis-induced repetitive myocardial injury results in global and segmental reduction in systolic cardiac function. Clin J Am Soc Nephrol. 2009;4(12):1925–31. 10.2215/CJN.04470709. 19808220 PMC2798881

[6] Gross ML, Ritz E. Non-coronary heart disease in dialysis patients: hypertrophy and fibrosis in the cardiomyopathy of Uremia—beyond coronary heart disease. Semin Dial. 2008;21(4):308–18. 10.1111/j.1525-139X.2008.00454.x. 18627569

[7] Nguyen NN, Van Duong P, Ngoc Mai TH, Vo NH, Luong DK, Ngo TH. Left ventricular Mass index in end-Stage renal disease patients during hemodialysis and continuous ambulatory peritoneal dialysis. Int J Clin practiceInt J Clin Pract. 2023;2023:1–7. 10.1155/2023/8816478. PMC10728365 38115951

[8] Han BG, Pak D, Kim JS, Sohn Y. The moderating effect of fluid overload on the relationship between the augmentation index and left ventricular diastolic function in patients with CKD. Sci Rep. 2024;14(1):480. 10.1038/s41598-023-50746-5. 38177252 PMC10767097

[9] Elsayed E, Farag YMK, Ravi KS, Chertow GM, Mc Causland FR. Association of changes in vector length with changes in left ventricular Mass among patients on maintenance hemodialysis: a secondary analysis of the frequent hemodialysis network daily trial. Kidney360. 2024;5(6):870–76. 10.34067/KID.0000000000000443. 38656312 PMC11219120

[10] Christa M, Smyth B, Mayne KJ, Stuard S, Canaud B, Genser B, et al. Increased risk of cardiac death with cumulative exposure to fluid overload and dialysate sodium ≤138 mmol/l in hemodialysis patients. Clin Kidney J. 2025;18(10):sfaf259. 10.1093/ckj/sfaf259. PMC12541371 41133181

[11] Holmberg B, Stegmayr BG. Cardiovascular conditions in hemodialysis patients may be worsened by extensive interdialytic weight gain. Hemodial Int. 2009;13(1):27–31. 10.1111/j.1542-4758.2009.00335.x. 19210274

[12] Westenbrink BD, Hovinga TK, Kloppenburg WD, Veeger NJ, Janssen WM. B-type natriuretic peptide and interdialytic fluid retention are independent and incremental predictors of mortality in hemodialysis patients. CN. 2011;76(11):373–79. 10.5414/CN106858. 22000557

[13] Saran R, Bragg-Gresham JL, Levin NW, Twardowski ZJ, Wizemann V, Saito A, et al. Longer treatment time and slower ultrafiltration in hemodialysis: associations with reduced mortality in the DOPPS. Kidney Int. 2006;69(7):1222–28. 10.1038/sj.ki.5000186. 16609686

[14] Movilli E, Gaggia P, Zubani R, Camerini C, Vizzardi V, Parrinello G, et al. Association between high ultrafiltration rates and mortality in uraemic patients on regular haemodialysis. A 5-year prospective observational multicentre study. Nephrol Dial Transpl. 2007;22(12):3547–52. 10.1093/ndt/gfm466. 17890254

[15] Flythe JE, Kimmel SE, Brunelli SM. Rapid fluid removal during dialysis is associated with cardiovascular morbidity and mortality. Kidney Int. 2011;79(2):250–57. 10.1038/ki.2010.383. 20927040 PMC3091945

[16] Assimon MM, Wenger JB, Wang L, Flythe JE. Ultrafiltration rate and mortality in maintenance hemodialysis patients. Am J Kidney Dis. 2016;68(6):911–22. 10.1053/j.ajkd.2016.06.020. 27575009 PMC5123913

[17] Navarrete JE, Rajabalan A, Cobb J, Lea JP. Proportion of hemodialysis treatments with high ultrafiltration rate and the association with mortality. Kidney360. 2022;3, 3(8):1359–66. 10.34067/KID.0001322022. PMC9416834 36176655

[18] Jefferies HJ, Virk B, Schiller B, Moran J, McIntyre CW. Frequent hemodialysis schedules are associated with reduced levels of dialysis-induced cardiac injury (myocardial stunning). Clin J Am Soc Nephrol. 2011;6(6):1326–32. 10.2215/CJN.05200610. 21597028 PMC3109928

[19] Burton JO, Jefferies HJ, Selby NM, McIntyre CW. Hemodialysis-induced cardiac injury: determinants and associated outcomes. Clin J Am Soc Nephrol. 2009;4(5):914–20. 10.2215/CJN.03900808. 19357245 PMC2676185

[20] Mahmood U, Johnson DW, Fahim MA. Cardiac biomarkers in dialysis. Aims Genet. 2017;4(1):1–20. 10.3934/genet.2017.1.1. 31435501 PMC6690238

[21] Yamashita K, Mizuiri S, Nishizawa Y, Shigemoto K, Doi S, Masaki T. Addition of novel biomarkers for predicting all-cause and cardiovascular mortality in prevalent hemodialysis patients. Ther Apher Dial. 2018;22(1):31–39. 10.1111/1744-9987.12593. 28971590

[22] Kawagoe C, Sato Y, Toida T, Nakagawa H, Yamashita Y, Fukuda A, et al. N-terminal-pro-B-type-natriuretic peptide associated with 2-year mortality from both cardiovascular and non-cardiovascular origins in prevalent chronic hemodialysis patients. Ren Fail. 2018;40(1):127–34. 10.1080/0886022X.2018.1437047. 29457529 PMC6014467

[23] Eriguchi M, Tsuruya K, Lopes M, Bieber B, McCullough K, Pecoits-Filho R, et al. Routinely measured cardiac troponin I and N-terminal pro-B-type natriuretic peptide as predictors of mortality in haemodialysis patients. ESC Heart Fail. 2022;9(2):1138–51. 10.1002/ehf2.13784. 35026869 PMC8934949

[24] Daniels LB, Maisel AS. Natriuretic peptides. J Am Coll Cardiol. 2007;50(25):2357–68. 10.1016/j.jacc.2007.09.021. 18154959

[25] Chaulin AM. Cardiac troponins metabolism: from biochemical mechanisms to clinical practice (literature review). IJMS. 2021;22(20):10928. 10.3390/ijms222010928. 34681585 PMC8535601

[26] Laveborn E, Lindmark K, Skagerlind M, Stegmayr B. NT-proBNP and troponin T levels differ after haemodialysis with a low versus high flux membrane. Int J Artif Organs. 2015;38(2):69–75. 10.5301/ijao.5000387. 25744196

[27] Goto J, Forsberg U, Jonsson P, Matsuda K, Nilsson B, Nilsson Ekdahl K, et al. Interdialytic weight gain of less than 2.5% seems to limit cardiac damage during hemodialysis. Int J Artif Organs. 2021;44(8):539–50. 10.1177/0391398820981385. 33339470 PMC8366174

[28] Pimenta J, Sampaio F, Martins P, Carvalho B, Rocha-Gonçalves F, Ferreira A, et al. Aminoterminal B-type natriuretic peptide (NT-proBNP) in end-stage renal failure patients on regular hemodialysis: does it have diagnostic and prognostic implications? Nephron Clin Pract. 2009;111(3):c182–8. 10.1159/000199458. 19194108

[29] Neumann JT, Twerenbold R, Ojeda F, Sorensen NA, Chapman AR, Shah ASV, et al. Application of high-sensitivity troponin in suspected myocardial infarction. N Engl J Med. 2019;380(26):2529–40. 10.1056/NEJMoa1803377. 31242362

[30] Goto J, Ott M, Stegmayr B. Myocardial markers are highly altered by higher rates of fluid removal during hemodialysis. Hemodial Int. 2024;28(1):17–23. 10.1111/hdi.13124. 37875435

[31] Schneditz D, Putz-Bankuti C, Ribitsch W, Schilcher G. Correction of plasma concentrations for effects of hemoconcentration or hemodilution. Asaio J. 2012;58(2):160–62. 10.1097/MAT.0b013e318243660f. 22370687

[32] Sveric KM, Cansız B, Winkler A, Ulbrich S, Ende G, Heidrich F, et al. Accuracy of Devereux and Teichholz formulas for left ventricular mass calculation in different geometric patterns: comparison with cardiac magnetic resonance imaging. Sci Rep. 2023;13(1):14089. 10.1038/s41598-023-41020-9. 37640771 PMC10462733

[33] Selby NM, McIntyre CW. The acute cardiac effects of dialysis. Semin Dial. 2007;20(3):220–28. 10.1111/j.1525-139X.2007.00281.x. 17555488

[34] Bos WJW, Bruin S, van Olden RW, Keur I, Wesseling KH, Westerhof N, et al. Cardiac and hemodynamic effects of hemodialysis and ultrafiltration. Am J Kidney Dis. 2000;35(5):819–26. 10.1016/S0272-6386(00)70250-2. 10793014

[35] Fernandez-Prado R, Pena-Esparragoza JK, Santos-Sanchez-Rey B, Pereira M, Avello A, Goma-Garces E, et al. Ultrafiltration rate adjusted to body weight and mortality in hemodialysis patients. Nefrologí(Engl Ed). 2021;41(4):426–35. 10.1016/j.nefroe.2021.10.005. 36165111

[36] Raimann JG, Wang Y, Mermelstein A, Kotanko P, Daugirdas JT. Ultrafiltration rate thresholds associated with increased mortality risk in Hemodialysis, unscaled or scaled to body size. Kidney Int Rep. 2022;7(7):1585–93. 10.1016/j.ekir.2022.04.079. 35812299 PMC9263411

[37] Yang LJ, Chao YL, Kuo IC, Niu SW, Hung CC, Chiu YW, et al. High ultrafiltration rate is associated with increased all-cause mortality in incident hemodialysis patients with a high Cardiothoracic ratio. J Pers Med. 2022;12(12):2059. 10.3390/jpm12122059. 36556279 PMC9786000

[38] Kanbay M, Ertuglu LA, Afsar B, Ozdogan E, Siriopol D, Covic A, et al. An update review of intradialytic hypotension: concept, risk factors, clinical implications and management. Clin Kidney J. 2020;13(6):981–93. 10.1093/ckj/sfaa078. 33391741 PMC7769545

[39] Charra B, Terrat JC, Vanel T, Chazot C, Jean G, Hurot JM, et al. Long thrice weekly hemodialysis: the Tassin experience. Int J Artif Organs. 2004;27(4):265–83. 10.1177/039139880402700403. 15163061

[40] Group FHNT, Chertow GM, Levin NW, Beck GJ, Depner TA, Eggers PW, et al. In-center hemodialysis six times per week versus three times per week. N Engl J Med. 2010;363(24):2287–300. 21091062 10.1056/NEJMoa1001593 PMC3042140

[41] Rocco MV, Lockridge RS Jr, Beck GJ, Eggers PW, Gassman JJ, Greene T, et al. The effects of frequent nocturnal home hemodialysis: the frequent hemodialysis network nocturnal trial. Kidney Int. 2011;80(10):1080–91. 10.1038/ki.2011.213. 21775973 PMC3569086

[42] Avila E, Sepulveda RA, Retamal J, Hachim D. Biocompatibility in hemodialysis: artificial membrane and human blood interactions. BMC Nephrol. 2025;26(1):482. 10.1186/s12882-025-04401-y. 40846913 PMC12374333

[43] Forsberg U, Jonsson P, Stegmayr B. The Emboless® venous chamber efficiently reduces air bubbles: a randomized study of chronic hemodialysis patients. Clin Kidney J. 2024;17(11):sfae323. 10.1093/ckj/sfae323. PMC11579606 39574541

[44] Bossola M, Mariani I, Strizzi CT, Piccinni CP, Di Stasio E. How to limit interdialytic weight gain in patients on maintenance hemodialysis: state of the art and perspectives. J Clin Med. 2025;14(6):1846. 10.3390/jcm14061846. 40142654 PMC11942859

[45] Yang WL, Fahim M, Johnson DW. Pathophysiology and significance of natriuretic peptides in patients with end-stage kidney disease. Clin Biochem. 2020;83:1–11. 10.1016/j.clinbiochem.2020.05.013. 32511964

[46] Arnadottir Á, Pedersen S, Bo Hasselbalch R, Goetze JP, Friis-Hansen LJ, Bloch-Munster AM, et al. Temporal release of high-sensitivity cardiac troponin T and I and copeptin after brief induced coronary artery balloon occlusion in humans. Circulation. 2021;143(11):1095–104. 10.1161/CIRCULATIONAHA.120.046574. 33297742

[47] Unlu S, Sahinarslan A, Sezenoz B, Uludag OM, Gokalp G, Seckin Ö, et al. High-sensitive troponin T increase after hemodialysis is associated with left ventricular global longitudinal strain and ultrafiltration rate. Cardiol J. 2020;27(4):376–83. 10.5603/CJ.a2018.0118. 30338843 PMC8016008

[48] Liebetrau C, Gaede L, Dorr O, Troidl C, Voss S, Hoffmann J, et al. Release kinetics of N-terminal pro-B-type natriuretic peptide in a clinical model of acute myocardial infarction. Clinica (Rome) Acta. 2014;429:34–37. 10.1016/j.cca.2013.11.017. 24291058

