# Supplementary material for: Effect of reduced ultrafiltration rate on the cardiac biomarkers NT-proBNP and troponin T: a prospective crossover study
Source: BMC Nephrol. 2026 Sep 16;27:547. doi: 10.1186/s12882-026-05364-4 (PMC13587309; doi:10.1186/s12882-026-05364-4)
Supplement: Supplementary file 1 [file 12882_2026_5364_MOESM1_ESM.docx]

**Supplement Item 1**

Optimal treatment time

t=IDWG/0.6

Minimal treatment time

t=IDWG _treatment2_/(ultrafiltration rate _treatment 1_*0.8)

Definitions

IDWG=((weight before treatment-target weight)/target weight)*100

Time in hours

Weight in kg

**Supplement Table 1 Estimated marginal means for the interaction between ultrafiltration rate and time of measurement for logNT-proBNP**.

Estimated marginal means derived from linear mixed models for log-transformed NT-proBNP values, with treatment (0=short vs. 1=prolonged session), timepoint (0=baseline, 1=180 min, 2=end), and their interaction as fixed effects, and a random intercept for patients.

**Supplement Table 2 Estimated marginal means for the interaction between ultrafiltration rate and time of measurement for logTnT**

Estimated marginal means derived from linear mixed models for log-transformed TnT values, with treatment (0=short vs. 1=prolonged session), timepoint (0=baseline, 1=180 min, 2=end), and their interaction as fixed effects, and a random intercept for patients.

| **Supplement Table 3 Difference in cardiac biomarkers unadjusted for haemoconcentration** | | | | | |
| --- | --- | --- | --- | --- | --- |
|  | **Standard session** | | **Prolonged session** | |  |
|  | Median | IQR | Median | IQR | p |
| NT-proBNP at start of treatment (ng/l) | 5322.0 | 22153.0 | 4582.0 | 24642.0 | 0.789 |
| NT-proBNP after 180 minutes of treatment (ng/l) | 7064.0 | 26810.0 | 5905.0 | 27515.0 | 0.130 |
| ∆NT-proBNP after 180 minutes of treatment (ng/l) | 1259.5 | 4369.0 | 1213.5 | 3176.0 | 0.003 |
| NT-proBNP at end of treatment (ng/l) | 7412.5 | 29202.0 | 6555.0 | 29254.0 | 0.406 |
| ∆NT-proBNP at end of treatment (ng/l) | 2218.5 | 6364.0 | 1990.0 | 6348.3 | 0.453 |
|  |  |  |  |  |  |
| Troponin T at start of treatment (ng/l) | 70.0 | 44.0 | 69.0 | 49.0 | 0.322 |
| Troponin T after 180 minutes of treatment (ng/l) | 77.0 | 50.0 | 78.0 | 61.0 | 0.633 |
| ∆Troponin T after 180 minutes of treatment (ng/l) | 8.0 | 6.0 | 7.0 | 7.0 | 0.110 |
| Troponin T at end of treatment (ng/l) | 79.0 | 53.0 | 81.0 | 58.0 | 0.410 |
| ∆Troponin T at end of treatment (ng/l) | 11.0 | 9.5 | 14.0 | 10.5 | 0.934 |

| **Supplement table 4 Echocardiographic findings** | | |
| --- | --- | --- |
|  | n | % |
| **Aortic regurgitation** |  |  |
| No | 13 | 68.4 |
| Mild | 4 | 21.1 |
| Moderate | 0 | 0 |
| Severe | 0 | 0 |
| Not commented/missing data | 2 | 10.5 |
| **Aortic stenosis** |  |  |
| No | 16 | 84.2 |
| Mild | 1 | 5.3 |
| Moderate | 1 | 5.3 |
| Severe | 0 | 0 |
| Not commented/missing data | 1 | 5.3 |
| **Mitral regurgitation** |  |  |
| No | 10 | 52.6 |
| Mild | 6 | 31.6 |
| Moderate | 2 | 10.5 |
| Severe | 1 | 5.3 |
| Not commented/missing data | 0 | 0 |
| **Diastolic dysfunction** |  |  |
| No | 8 | 42.1 |
| Mild | 3 | 15.8 |
| Moderate | 1 | 5.3 |
| Severe | 0 | 0 |
| Not commented/missing data | 7 | 36.9 |
| **Left ventricular ejection fraction** | |  |
| >50% | 14 | 73.7 |
| 41-49% | 2 | 10.5 |
| <40% | 3 | 15.8 |
|  |  |  |
| **Numerical parameters (n=14)** | Mean | SD |
|  |  |  |
| LVEDD (mm) | 50.3 | 8.2 |
| IVSD (mm) | 13.3 | 2.9 |
| PWD (mm) | 9.6 | 1.9 |
| LVM (g) | 169.4 | 38.1 |
| LVMI (g/m^2^) | 93.7 | 20.7 |

Echocardiographic findings on echocardiographs done under stable conditions within one year of study. LVEDD: left ventricular end diastolic diameter. IVSD Intraventricular septum diameter. PWD posterior wall diameter. LVM Left ventricular mass. LVMI Left ventricular mass indexed to body surface area.

**Supplement Table 5**

|  | | Age | Vintage (months) | LVMI (g/m^2^) | QRS (mm) | IDWG (%) | NT-proBNP (ng/l) before session | TnT (ng/l) before session | ∆NT-proBNP (ng/l) ordinary session | ∆TnT (ng/l) ordinary session | ∆NT-proBNP (ng/l) prolonged session | ∆TnT (ng/l) prolonged session |
| --- | --- | --- | --- | --- | --- | --- | --- | --- | --- | --- | --- | --- |
| Age | Spearman´s rho |  | 0.228 | 0.156 | 0.294 | -0.229 | 0.477^**^ | 0.396^*^ | 0.437^**^ | 0.213 | 0.371^*^ | 0.105 |
|  | p |  | 0.151 | 0.593 | 0.066 | 0.151 | 0.002 | 0.023 | 0.005 | 0.235 | 0.018 | 0.561 |
|  | N |  | 41 | 14 | 40 | 41 | 41 | 33 | 40 | 33 | 40 | 33 |
| Vintage (months) | Spearman´s rho |  |  | -0.116 | 0.006 | -0.146 | 0.082 | -0.061 | 0.081 | 0.005 | -0.057 | -0.002 |
|  | p |  |  | 0.692 | 0.970 | 0.362 | 0.612 | 0.735 | 0.621 | 0.976 | 0.725 | 0.993 |
|  | N |  |  | 14 | 40 | 41 | 41 | 33 | 40 | 33 | 40 | 33 |
| LVMI (g/m^2^) | Spearman´s rho |  |  |  | 0.315 | -0.433 | 0.143 | 0.418 | 0.253 | 0.269 | 0.220 | 0.478 |
|  | p |  |  |  | 0.272 | 0.122 | 0.626 | 0.155 | 0.405 | 0.374 | 0.471 | 0.098 |
|  | N |  |  |  | 14 | 14 | 14 | 13 | 13 | 13 | 13 | 13 |
| QRS (mm) | Spearman´s rho |  |  |  |  | -0.140 | 0.396^*^ | 0.180 | 0.209 | -0.005 | 0.234 | 0.189 |
|  | p |  |  |  |  | 0.390 | 0.011 | 0.316 | 0.203 | 0.980 | 0.151 | 0.291 |
|  | N |  |  |  |  | 40 | 40 | 33 | 39 | 33 | 39 | 33 |
| IDWG (%) | Spearman´s rho |  |  |  |  |  | 0.140 | -0.044 | 0.216 | 0.144 | 0.295 | 0.001 |
|  | p |  |  |  |  |  | 0.382 | 0.807 | 0.182 | 0.425 | 0.065 | 0.994 |
|  | N |  |  |  |  |  | 41 | 33 | 40 | 33 | 40 | 33 |
| NT-proBNP (ng/l) before session | Spearman´s rho |  |  |  |  |  |  | 0.384^*^ | 0.831^**^ | 0.186 | 0.850^**^ | 0.328 |
|  | p |  |  |  |  |  |  | 0.027 | <0.001 | 0.300 | <0.001 | 0.062 |
|  | N |  |  |  |  |  |  | 33 | 40 | 33 | 40 | 33 |
| TnT (ng/l) before session | Spearman´s rho |  |  |  |  |  |  |  | 0.371^*^ | 0.501^**^ | 0.387^*^ | .407^*^ |
|  | p |  |  |  |  |  |  |  | 0.033 | 0.003 | 0.026 | 0.019 |
|  | N |  |  |  |  |  |  |  | 33 | 33 | 33 | 33 |

**Supplement Figure 1**

Individual values of NT-proBNP at different timepoints during haemodialysis sessions. Values at 180 minutes and end of treatment adjusted for haemoconcentration.

**Supplement figure 2**

Individual values of Troponin T at different timepoints during haemodialysis sessions. Values at 180 minutes and end of treatment adjusted for haemoconcentration.
